# *Lacticaseibacillus paracasei* LPB27: Effectively Enhances the Immune System by Improving the Intestinal Environment

**DOI:** 10.3390/foods15173033

**Published:** 2026-08-27

**Authors:** Shaoshi Zhang, Yueyi Guo, Jiangyan Zhao, Jian Zhao, Yu Guo, Wenjie Yan

**Affiliations:** 1 Beijing Key Laboratory of BioActive Substances and Functional Food, College of Biochemical Engineering, Beijing Union University, Beijing 100023, China; 18810839157@163.com (S.Z.); guoyueyits@sina.com (Y.G.); jiangyanmail@263.net (J.Z.); z386681913@sina.com (J.Z.); gyanling9408@sohu.com (Y.G.); 2 Functional Testing Center of Health Food, College of Applied Arts and Sciences, Beijing Union University, Beijing 100191, China

**Keywords:** *Lacticaseibacillus paracasei* LPB27, immunity, intestinal flora, metabolic pathway, immunoglobulin

## Abstract

*Lacticaseibacillus paracasei* LPB27 is a novel probiotic isolated from the intestines of healthy infants and young children. In this study, healthy mice were used as a model to systematically investigate the immune regulatory effects of LPB27 mediated by the microbiota–gut–immune axis, with a focus on evaluating its immune-enhancing potential. The safety evaluation showed that, at a dose of 0.50 g/kg/day, LPB27 caused no observable toxicity or adverse reactions. The evaluation of immune function indicated that in terms of cellular immunity, high-dose LPB27 increased the delayed-type hypersensitivity response and lymphocyte proliferation by 69% and 35%, respectively; in terms of humoral immunity, the number of hemolytic plaques and hemolysin titer increased by 42% and 36%, respectively; in terms of innate immunity, the carbon clearance capacity, CRBC phagocytosis rate, and macrophage phagocytic index increased by 13%, 33%, and 66%, respectively. Serum IgA, IgG, and IL-6 levels were significantly elevated in the high-dose group, whereas TNF-α was significantly reduced. Overall, LPB27 supplementation enhanced multiple immune indicators in mice. By comparing the gut microbiota composition at different time points, the study found that continuous intake of LPB27 was associated with an enrichment of multiple beneficial bacterial genera, including *Akkermansia*, *Lactobacillus*, *Muribaculum*, and *Lachnospiraceae_NK4A136_group*, together with a reduction of potentially pathogenic taxa, indicating an overall improvement in the intestinal microbial ecology. Changes in the abundance of these beneficial taxa were positively correlated with the predicted activation of metabolic pathways related to the synthesis of immune-related proteins. These findings support the further development of LPB27 as an immune-regulating ingredient.

## 1. Introduction

The immune system is a critical defense mechanism that protects the body against pathogens and maintains internal homeostasis, and its functional status closely influences overall health and the onset and progression of numerous diseases [1,2,3].

The microbiota–gut–immune axis [4] describes the bidirectional communication among the gut microbiota, the intestinal epithelium, and the host immune system. Gut-resident microbes shape mucosal and systemic immunity through microbial metabolites and structural components, while immune status in turn shapes the composition and function of the microbiota. This framework has expanded the study of probiotic immunomodulation beyond traditional nutritional science and established it as a focus of interdisciplinary research at the intersection of immunometabolism and systems biology [5]. *Lacticaseibacillus paracasei*, a representative Gram-positive probiotic, displays surface-layer proteins, including the S-layer protein SlpA and mucus-binding proteins (MUBs), and harbors cell-wall components such as peptidoglycan and exopolysaccharides. These surface and structural components can interact with host pattern recognition receptors (PRRs) to modulate downstream immune signaling pathways [6,7,8].

*Lacticaseibacillus paracasei* LPB27 is a newly identified probiotic strain isolated from the intestinal tract of healthy Chinese infants. Strains isolated from the intestinal tract of healthy populations are one of the prioritized primary screening sources for probiotic candidates in the direction of immune enhancement. However, no standardized evaluation of its immunomodulatory effects has yet been conducted [9]. The Chinese national guideline Functional Inspection and Evaluation Methods for Health Food (2023 Edition), which includes the Test Methods for Enhancing Immunity, specifies standardized, regulatory-relevant assays (e.g., delayed-type hypersensitivity, hemolytic plaque formation, carbon clearance, and NK cell activity) for the functional assessment of health foods in China. This study therefore followed that guideline to evaluate the immunomodulatory mechanisms of *L. paracasei* LPB27 in a healthy mouse model, providing a theoretical basis for product development.

## 2. Materials and Methods

### 2.1. Materials and Reagents

*Lacticaseibacillus paracasei* LPB27, a strain derived from the intestines of infants and young children, is provided by BY-HEALTH CO., LTD. (Zhuhai, China) as a purified lyophilized powder with a live bacterial count of 1.0 × 10^11^ CFU/g. RPMI 1640 culture medium (catalog no. 12633020, Gibco, Waltham, MA, USA) and fetal bovine serum (catalog no. 10099-141, Gibco/Thermo Fisher, Waltham, MA, USA) were purchased from Gibco (USA). Giemsa stain (catalog no. G1015, Solarbio, Beijing, China) was acquired from Beijing Solarbio Science & Technology Co., Ltd. (Beijing, China). Mouse interferon-γ (IFN-γ; catalog no. ml002277), tumor necrosis factor-α (TNF-α; catalog no. ml002095), interleukin-6 (IL-6; catalog no. ml063159), immunoglobulin A (IgA; catalog no. ml037602), and immunoglobulin G (IgG; catalog no. ml037601) enzyme-linked immunosorbent assay (ELISA) kits were obtained from Shanghai Enzyme-linked Biotechnology Co., Ltd. (Shanghai, China). All other reagents and solvents used in this study were of analytical grade.

### 2.2. Animals and Experimental Design

#### 2.2.1. Establishment of Animal Model

A total of 192 male specific-pathogen-free (SPF) Kunming (KM) mice, weighing 18–22 g and aged 6–8 weeks, were obtained from Beijing Huafukang Bioscience Co., Ltd. (License No.: SCXK(Jing)2019-0008). The animals were housed in a specific-pathogen-free (SPF) grade animal facility at the Health Food Function Testing Center of Beijing Union University (License No.: SYXK(Jing)2017-0038). The experimental protocol was approved by the Animal Experimental Ethics Committee of the Functional Testing Center of Health Food, College of Applied Arts and Sciences, Beijing Union University (Approval No.: 20240903), in accordance with the Chinese national standard GB/T 35892-2018 “Laboratory Animal—Guideline for Ethical Review of Animal Welfare”.

Following a 7-day acclimatization period, initial body weights were recorded, and the mice were randomly assigned to 16 experimental groups (12 animals per group) using a blocked randomization method stratified by body weight, ensuring balanced baseline body weights across groups. The 16 groups were then assigned to four separate batches (4 groups per batch) by complete randomization for the experimental measurements. The recommended daily dose of *Lacticaseibacillus paracasei* LPB27 probiotic powder for humans is 1 g per 60 kg of body weight (BW). Based on this reference, the experimental doses were set at 5-, 10-, and 30-fold the human-equivalent dose, corresponding to 0.08, 0.17, and 0.50 g/kg/day for the low-, medium-, and high-dose groups, respectively. The LPB27 was administered orally once daily for 31 consecutive days, after which a range of biological parameters were evaluated. The gavage volume was standardized to 10 mL/kg of body weight. A blank control group (0 g/kg BW) was included, in which sterile water was administered using the same gavage volume as that applied to the treatment groups. All dose groups were maintained on a standard maintenance diet throughout the experimental period.

#### 2.2.2. Experimental Design

**Scheme 1:**
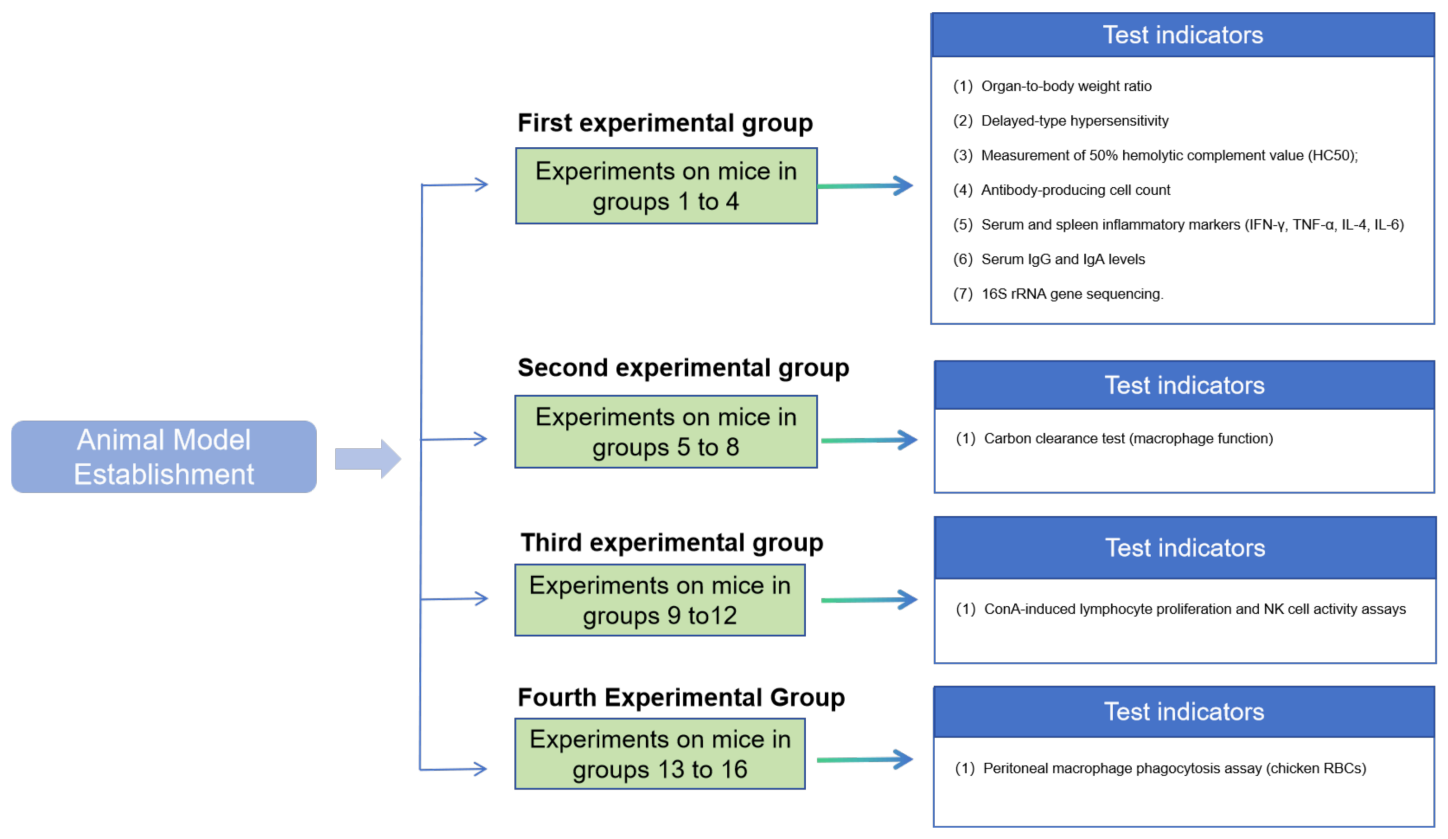
Schematic overview of the experimental design. Note: According to the administration regimen described in Section 2.2.1, each experimental group was divided into four dose groups (control, low, medium, and high), which were administered LPB27 for 31 consecutive days; a total of 192 mice were randomly assigned to 16 groups and further combined into four experimental groups for the different measurements.

As shown in Scheme 1.

Batch 1: This batch was designed to evaluate the organ-to-body weight ratio, perform delayed-type hypersensitivity tests, determine the 50% hemolytic complement value (HC_50_), and quantify antibody-producing cells. In addition, serum and spleen levels of inflammatory markers, including IFN-γ, TNF-α, IL-4, and IL-6, were analyzed. Serum immunoglobulin levels (IgG and IgA) were also measured. Fecal samples collected on days 15 and 31 were analyzed for short-chain fatty acid content and subjected to 16S rRNA gene sequencing.

Batch 2: This batch involved the carbon clearance test to assess macrophage function.

Batch 3: This batch included the ConA-induced lymphocyte proliferation assay and the natural killer (NK) cell activity assay.

Batch 4: This batch focused on the peritoneal macrophage phagocytosis assay using chicken red blood cells as target particles.

### 2.3. Experimental Methods

#### 2.3.1. Determination of Body Weight and Organ-to-Body Weight Ratios

The body weights of the mice were recorded before and after the experimental intervention to evaluate the dynamic effects of LPB27 on body weight regulation. On the day of sacrifice, the animals were weighed and subsequently euthanized by cervical dislocation. The spleen and thymus were rapidly excised following euthanasia. After removal of the fascial tissue from the organ surfaces, residual blood was absorbed using filter paper, and the organs were immediately weighed with precision. The spleen index (spleen weight/body weight) and thymus index (thymus weight/body weight) were subsequently calculated.

#### 2.3.2. ConA-Induced Mouse Lymphocyte Transformation Assay (MTT Method)

Spleen was aseptically ground into single-cell suspension in Hank’s, filtered (200-mesh), washed twice (Hank’s, 1000 rpm/10 min), resuspended in 1 mL complete medium, and adjusted to 3 × 10^6^ cells/mL [10]. It was added 1.0 mL/well to 24-well plate; experimental group received 75 µL ConA (catalog no. C8110, Solarbio, Beijing, China) (final 7.5 µg/mL), while control wells received an equal volume of culture medium without ConA to serve as the unstimulated control. It was incubated (5% CO_2_, 37 °C, 72 h); 4 h before the end, 0.7 mL supernatant/well was removed, and 0.7 mL serum-free RPMI 1640 + 50 µL MTT was added (catalog no. M8180, Solarbio, Beijing, China) (5 mg/mL); it was then incubated 4 h more. Next, 1.0 mL acidic isopropanol/well was added to dissolve crystals, and we measured OD at 570 nm. The lymphocyte proliferation capacity was expressed as the difference in OD between the ConA-stimulated and unstimulated control wells.

#### 2.3.3. Delayed-Type Hypersensitivity (DTH) Test

Defibrinated sheep blood was washed three times with saline, centrifuged at 2000 rpm for 10 min, and resuspended to prepare a 2% (*v*/*v*) suspension of sheep red blood cells (SRBCs) in saline [11]. Mice were sensitized via intraperitoneal (i.p.) administration of 0.2 mL SRBC suspension. Four days later, the thickness of the left hind paw was recorded. Subsequently, 20 µL of a 20% (*v*/*v*) SRBC suspension in saline was administered subcutaneously (s.c.) at the same anatomical site. Paw thickness was measured again 24 h after the injection. Each measurement was performed in triplicate, and the mean value was calculated. The intensity of delayed-type hypersensitivity (DTH) was evaluated based on the change in paw thickness (Δthickness) before and after antigenic challenge.

#### 2.3.4. Determination of Antibody-Generating Cell Count (Jerne Modified Slide Method)

Defibrinated sheep blood was washed three times with physiological saline [12]. Each mouse received 0.2 mL of a 2% (*v*/*v*) suspension of sheep red blood cells (SRBCs) via intraperitoneal injection. Five days after immunization, the mice were euthanized and their spleens were removed. Spleen cells were filtered through a 200-mesh sieve, centrifuged at 1000 rpm for 10 min, washed twice with Hank’s balanced salt solution (HBSS), and resuspended in 8.0 mL of HBSS. A 1% agarose solution was mixed with an equal volume of double-strength HBSS and divided into 0.5 mL aliquots. Then, 50 µL of a 10% (*v*/*v*) SRBC suspension and 8 µL of the splenic cell suspension were added to each aliquot. The mixture was vortexed thoroughly and spread onto adhesive-coated glass slides to prepare duplicate assay slides. After gelling at room temperature, the slides were incubated at 37 °C in a humidified 5% CO_2_ incubator for 1 h. Complement diluted 1:8 in SA buffer was then added, followed by incubation for 1.5 h at 37 °C. Hemolytic plaques were counted under a stereomicroscope and expressed as plaques per million splenic cells. Splenic cells from control mice (sterile water) were processed identically to serve as the negative control. A statistically significant increase in plaque count in the test group, compared with the control group, indicated a positive immune response.

#### 2.3.5. Determination of Median Hemolytic Complement Value

Defibrinated sheep blood was washed three times with saline. Mice received an intraperitoneal (i.p.) injection of 0.2 mL of a 2% SRBC suspension [13]. Five days later, blood was collected and serum separated by standing for 1 h and centrifugation at 3000 rpm for 10 min. The serum was diluted 300-fold with SA buffer, and 1.0 mL was added to a tube with 0.5 mL of a 10% SRBC suspension and 1.0 mL of complement diluted 1:8 in SA buffer. A control tube used SA buffer instead. The mixture was incubated at 37 °C for 15 min, then placed on ice to stop the reaction. Samples were centrifuged at 2000 rpm for 10 min. One milliliter of supernatant was mixed with 3.0 mL of hemoglobin diluent. For the standard, 0.25 mL of 10% SRBCs was diluted to 4.0 mL with diluent. OD was measured at 540 nm using the control as blank. Hemolysin activity was expressed as HC50, calculated as:

HC_50_ = (Sample OD/Median hemolytic OD of SRBCs) × Dilution factor.

#### 2.3.6. Mouse Carbon Clearance Test

Mice received a 4-fold diluted India ink solution via tail vein injection based on body weight, with timing starting immediately [13]. Blood (20 µL) was collected at 2 and 10 min post-injection from the inner canthus and added to 2.0 mL of 0.1% sodium carbonate (Na_2_CO_3_) solution. The optical density (OD) was measured at 600 nm using the Na_2_CO_3_ solution as the blank. After collection, the mice were euthanized and the liver and spleen were weighed. The carbon clearance capacity was expressed as the phagocytic index (α), calculated using the following formulas:

k = (log_10_ OD_1_ − log_10_ OD_2_)/(t_2_ − t_1_); α = [Body weight/(Liver weight + Spleen weight)] × k^(1/3).

#### 2.3.7. Phagocytosis of Chicken Red Blood Cells by Mouse Peritoneal Macrophages

Mice were injected intraperitoneally (i.p.) with 1 mL of a 20% (*v*/*v*) suspension of chicken erythrocytes in saline, prepared by centrifugation at 2000 rpm for 10 min [14]. Thirty minutes later, the mice were euthanized and fixed on a dissection board. The peritoneal cavity was lavaged with 2 mL of saline, and 1 mL of lavage fluid was collected. The lavage fluid was evenly distributed onto two glass slides and incubated for 30 min at 37 °C in a humidified chamber. After incubation, the slides were rinsed gently with saline, air-dried, fixed with a 1:1 mixture of acetone and methanol, stained with Giemsa stain prepared in phosphate buffer, rinsed thoroughly with distilled water, and air-dried again. A total of 100 macrophages per slide were counted under oil immersion microscopy, and macrophages that had engulfed at least one chicken red blood cell were scored as phagocytic. The phagocytosis indices were calculated as follows:

Phagocytic % = (Number of macrophages phagocytosing chicken red blood cells [CRBCs]/Total number of macrophages counted) × 100%; Phagocytic Index = Total number of phagocytosed CRBCs/Total number of macrophages counted.

Phagocytic % was arcsine-transformed: X = sin^−1^(√P), where P is the phagocytic percentage expressed as a decimal (e.g., 0.45 for 45%).

#### 2.3.8. Determination of NK Cell Activity

Three days before the assay, the mouse lymphoma YAC-1 target cells were subcultured, washed three times with Hank’s balanced salt solution, and adjusted to 4 × 10^5^ cells/mL [15]. Mouse spleens were processed into single-cell suspensions, washed, centrifuged, and treated for red blood cell lysis, then resuspended to 2 × 10^7^ cells/mL (an effector-to-target [E:T] ratio of 50:1). Target and effector cells were added to a 96-well plate containing natural and maximum release controls, and incubated for 4 h. After centrifugation, the supernatant was mixed with lactate dehydrogenase (LDH) substrate, the reaction was stopped, and OD was measured. NK activity was calculated and arcsine-transformed (X = sin − 1√P).

#### 2.3.9. Serum Assay for Inflammatory Markers and IgA/IgG Measurement

Sheep blood was washed three times with saline. Each mouse was immunized by intraperitoneal (i.p.) injection of 0.2 mL of a 2% (*v*/*v*) suspension of sheep red blood cells (SRBCs) in saline. Five days later, blood was collected into tubes, allowed to clot for approximately 1 h, centrifuged at 3000 rpm for 10 min, and the serum was collected. The concentrations of IFN-γ, TNF-α, IL-6, IgA, and IgG were determined using the corresponding commercial ELISA kits (catalog nos. ml002277, ml002095, ml063159, ml037602, and ml037601, respectively; Shanghai Enzyme-linked Biotechnology Co., Ltd., batch 2024-07), strictly following the manufacturer’s instructions [16]. In brief, standards and serum samples were added to the pre-coated microplate and incubated at 37 °C for 60 min. After five washes, horseradish peroxidase (HRP)-conjugated detection antibody was added and incubated at 37 °C for 60 min. Following another wash, substrate solutions A and B were added and the plate was incubated at 37 °C in the dark for 15 min for color development. The reaction was stopped by adding stop solution, and the absorbance was read at 450 nm using a microplate reader. Analyte concentrations were calculated from a four-parameter logistic standard curve and multiplied by the sample dilution factor to obtain the final concentrations.

#### 2.3.10. Determination of Short-Chain Fatty Acids

Standard solutions of acetic acid, propionic acid, isobutyric acid, and butyric acid were prepared [17]. After chromatographic analysis, a calibration curve of concentration versus peak area ratio was plotted. Mouse fecal samples (0.2 g) were weighed into microcentrifuge tubes, mixed with 0.25 mL of 25% phosphoric acid solution, and diluted to a final volume of 1.5 mL with acetone. After standing at room temperature for 10 min, 0.35 mL of the supernatant was transferred. This aliquot was then diluted to 1 mL with acetone. Following high-speed centrifugation (13,000× *g*, 10 min, 4 °C), 200 μL of the supernatant was collected for chromatographic analysis.

#### 2.3.11. Mouse Fecal Gut Microbiota Analysis

Fecal samples from six randomly selected mice per group were subjected to 16S rRNA gene sequencing. Fresh fecal samples were collected prior to euthanasia. Microbial DNA was extracted using a magnetic bead-based method, quantified using the Qubit assay, and subjected to PCR amplification. Purified PCR products were evaluated using an Agilent 2100 Bioanalyzer (Agilent Technologies, Santa Clara, CA, USA) and Illumina library quantification kits (Illumina, San Diego, CA, USA). Qualified libraries were diluted, pooled proportionally, denatured with NaOH, and sequenced on a NovaSeq 6000 platform (Illumina, San Diego, CA, USA) to generate 2 × 250 bp paired-end reads. Raw sequencing data underwent overlap merging, low-quality sequence filtering, and chimera removal to generate high-quality Clean Data. Clean Data were denoised using QIIME2 DADA2 [18,19] to correct PCR and sequencing errors, yielding Amplicon Sequence Variants (ASVs) and their corresponding abundance tables. Subsequent analyses included alpha- and beta-diversity assessments, taxonomic composition and differential abundance analysis, and functional composition and differential abundance analysis.

### 2.4. Data Statistics

Experimental data were analyzed using SPSS 26.0 software. All experimental data are presented as mean ± standard deviation (SD). The Shapiro–Wilk test was applied to evaluate the normality of raw data, and Levene’s test was used to examine homogeneity of variance. For datasets satisfying normal distribution and homogeneity of variance, one-way analysis of variance (ANOVA) was adopted for overall intergroup comparison, followed by Tukey’s post hoc test for multiple pairwise comparisons; for datasets with non-normal distribution or heterogeneous variance, the nonparametric Kruskal–Wallis H test was performed for overall intergroup comparison, with Dunn’s post hoc correction applied for subsequent pairwise comparisons. Graphs were generated using GraphPad Prism version 6. Statistical significance was defined as *p* < 0.05, *p* < 0.01 and *p* < 0.001, marked separately. All statistical approaches used for each index were annotated in figure legends and table footnotes correspondingly.

## 3. Results and Discussion

### 3.1. Effect of LPB27 on the Organ/Body Weight Ratio in Mice

**Figure 1:**
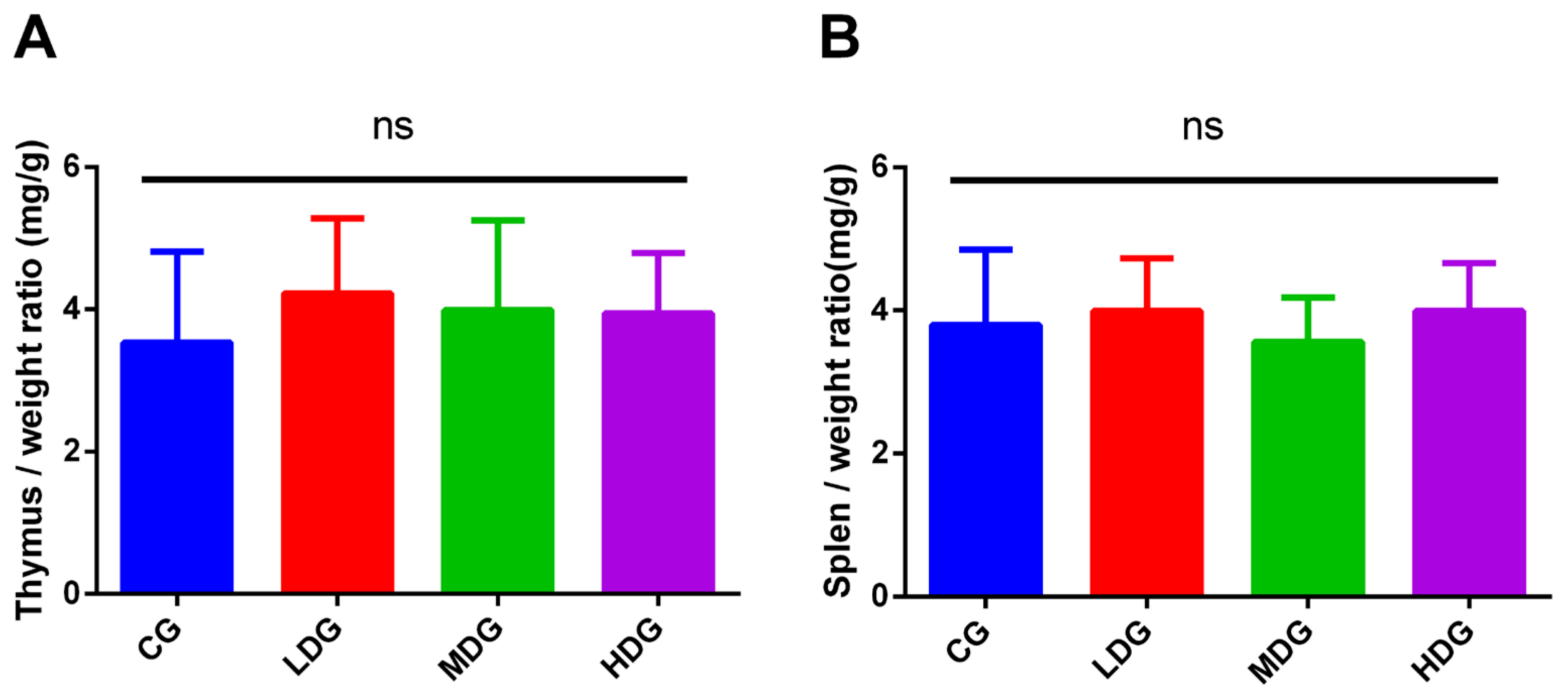
Organ/body weight ratio in mice. Note: Thymus/body weight ratio (**A**) and spleen/body weight ratio (**B**). CG (0 g/kg/day), LDG (0.08 g/kg/day), MDG (0.17 g/kg/day), HDG (0.50 g/kg/day). ns, not significant (*p* > 0.05). Data are expressed as mean ± SD (*n* = 12).

The organ-to-body weight ratio represents a critical parameter for the evaluation of immunotoxicity [20]. As shown in Figure 1, following 31 days of oral administration of varying doses of LPB27 to mice, no significant differences were observed among all groups. These results indicate that, under the experimental conditions of the present study, LPB27 does not raise safety concerns at any of the administered dose levels.

The absence of significant changes in the spleen-to-body and thymus-to-body weight ratios across all dose groups indicates that LPB27 is well tolerated and does not raise immunotoxicity concerns at any of the doses tested. These results provide a safety basis for the immunomodulatory evaluations presented in the following sections.

### 3.2. Effects of LPB27 on the Mouse Immune System

LPB27 administration modulated both adaptive and innate immune responses in mice. The effects on adaptive immunity were evaluated through cellular and humoral responses, and those on innate immunity through macrophage phagocytosis and NK cell activity.

#### 3.2.1. Effects of LPB27 on Adaptive Immunity in Mice

##### Cellular Immune Responses

**Figure 2:**
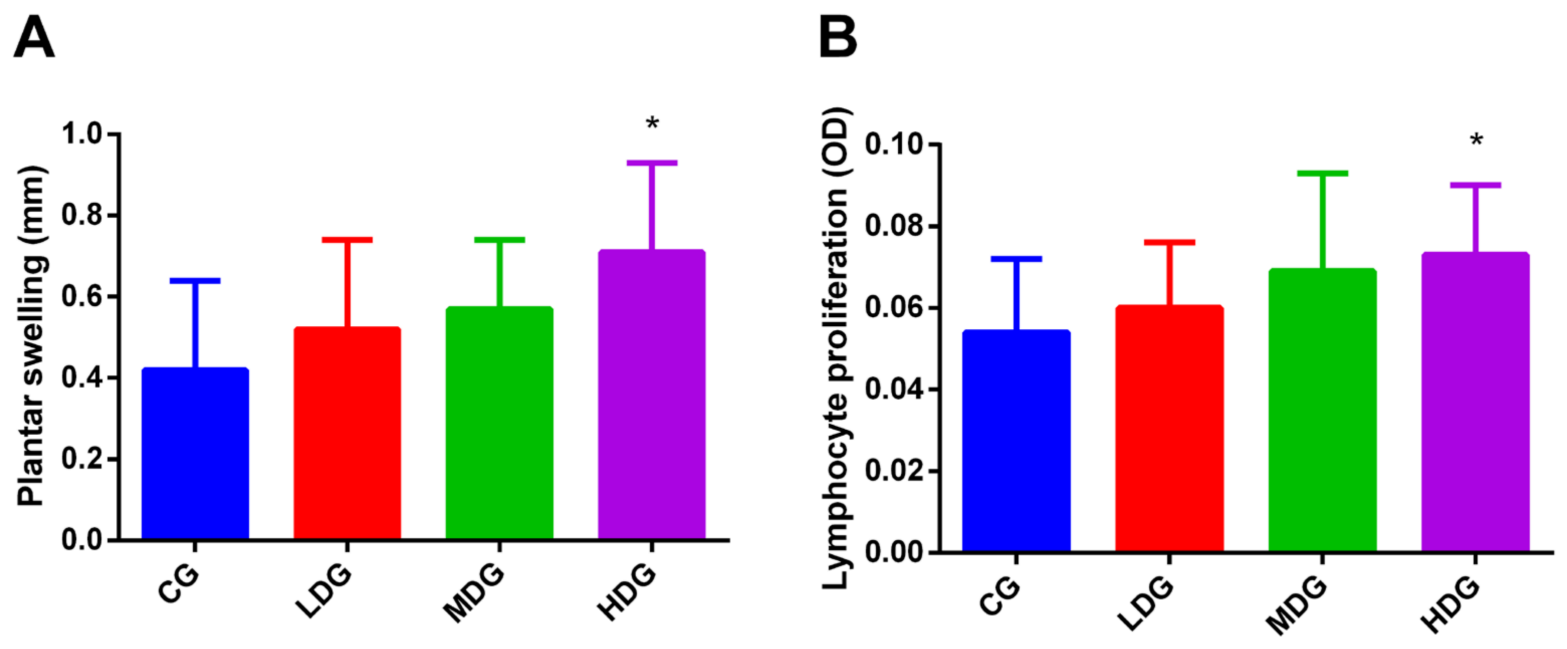
Effects of LPB27 dose on cellular immune indicators in mice. Note: Plantar swelling (**A**) and lymphocyte proliferation capacity (**B**). CG (0 g/kg/day), LDG (0.08 g/kg/day), MDG (0.17 g/kg/day), HDG (0.50 g/kg/day). * *p* < 0.05 vs. CG. Data are expressed as mean ± SD (*n* = 12).

Cellular immune function was assessed using the delayed-type hypersensitivity (DTH) response and the lymphocyte proliferation assay [21,22,23]. As shown in Figure 2, following 31 days of oral administration of LPB27 at varying doses to mice, footpad swelling and lymphocyte proliferation in the 0.50 g/kg/day (high-dose) group were significantly increased by 69% and 35%, respectively, compared with the control group. These findings demonstrate that LPB27 effectively enhances lymphocyte proliferation, thereby improving cellular immune function.

##### Humoral Immune Responses

**Figure 3:**
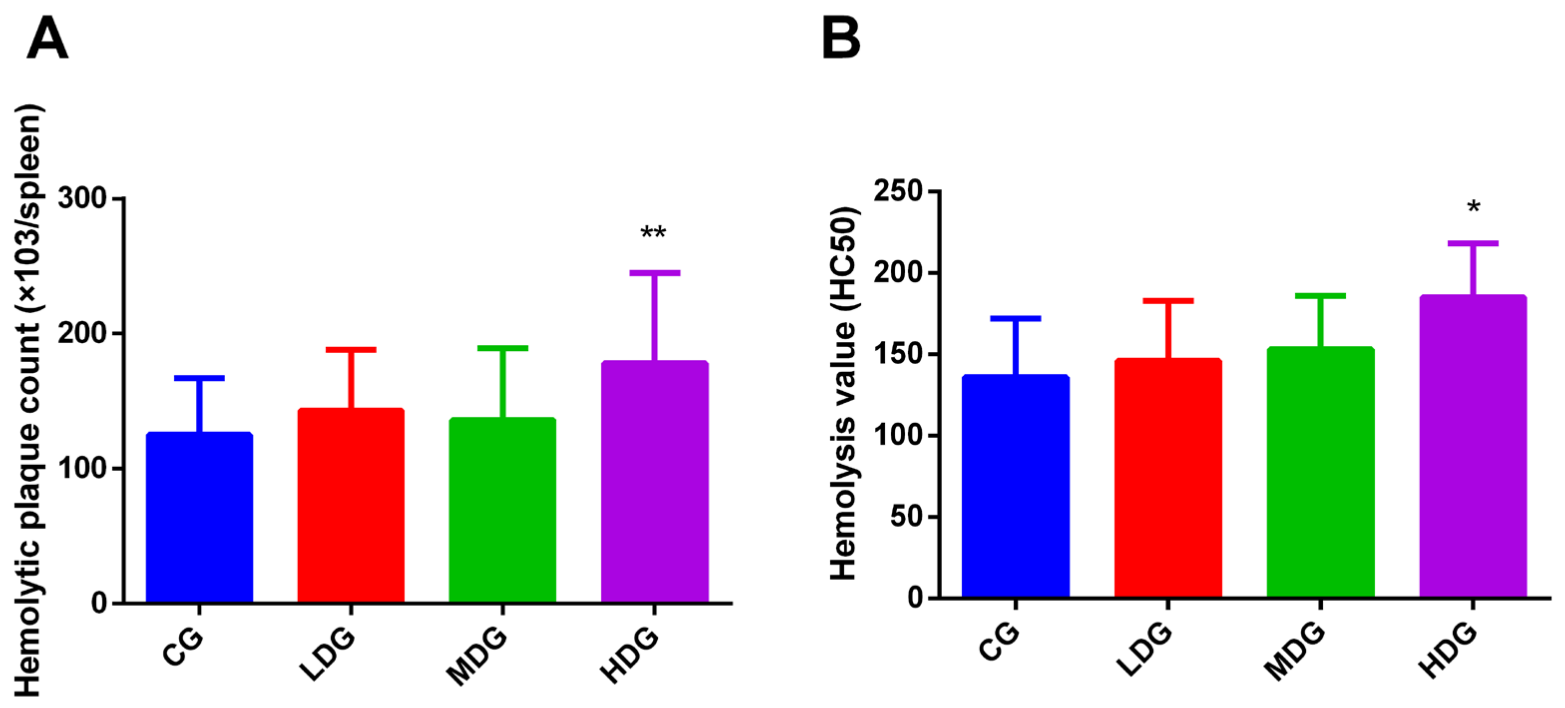
Effects of LPB27 dose on humoral immune indicators in mice. Note: Hemolytic plaque count (**A**) and hemolysis value/HC50 (**B**). CG (0 g/kg/day), LDG (0.08 g/kg/day), MDG (0.17 g/kg/day), HDG (0.50 g/kg/day). * *p* < 0.05, ** *p* < 0.01 vs. CG. Data are expressed as mean ± SD (*n* = 12).

Humoral immune function was evaluated using the hemolytic plaque assay and the HC50 value [24,25,26]. As shown in Figure 3, following 31 days of oral administration of LPB27 at varying doses to mice, the hemolytic plaque count and HC50 values in the 0.50 g/kg/day (high-dose) group were significantly increased by 42% and 36%, respectively, compared with the control group. The observed increase in plaque count demonstrates that LPB27 markedly enhances B lymphocyte proliferation, which is directly involved in antibody production. Furthermore, the elevation in HC50 values supports the conclusion that LPB27 effectively increases serum antibody titers, thereby enhancing the humoral immune response.

Together, these results demonstrate that LPB27 enhanced adaptive immunity in a dose-dependent manner, with the high-dose treatment (0.50 g/kg/day) significantly increasing delayed-type hypersensitivity, lymphocyte proliferation, hemolytic plaque counts, and the HC50 value. These findings are consistent with evidence that probiotic lactobacilli modulate host immunity through the microbiota–gut–immune axis [4,5] and with previous reports of the immunomodulatory activity of *L. paracasei* strains [6,7].

#### 3.2.2. Effects of LPB27 on Innate Immunity in Mice

##### Macrophage Phagocytosis

**Figure 4:**
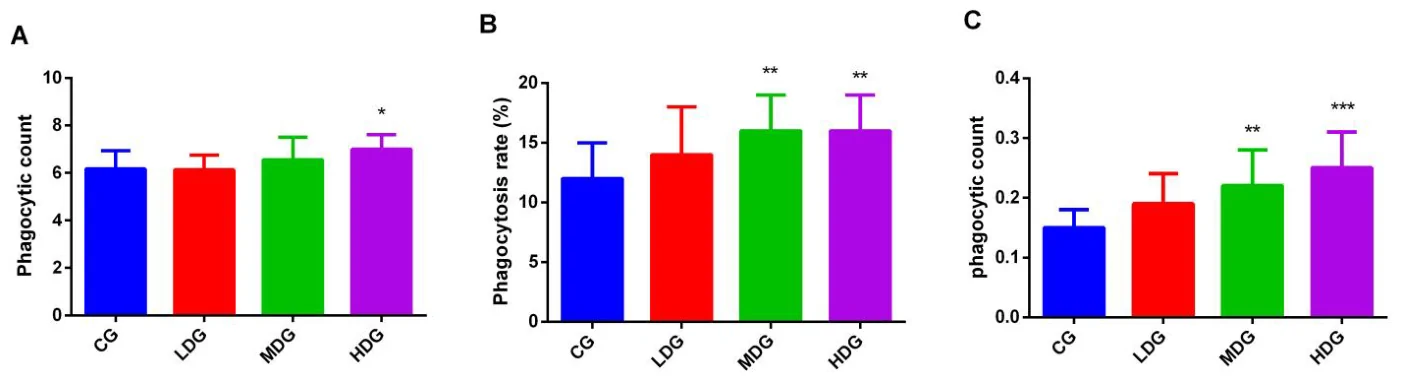
Phagocytosis function of mouse monocytes and macrophages. Note: Carbon clearance capacity (**A**), phagocytosis rate of chicken red blood cells (**B**), and phagocytic index (**C**). CG (0 g/kg/day), LDG (0.08 g/kg/day), MDG (0.17 g/kg/day), HDG (0.50 g/kg/day). * *p* < 0.05, ** *p* < 0.01, *** *p* < 0.001 vs. CG. Data are expressed as mean ± SD (*n* = 12).

To further investigate alterations in nonspecific immune function, carbon clearance assays and peritoneal macrophage phagocytosis of CRBCs were conducted [27]. As shown in Figure 4, following 31 days of oral administration of LPB27 at varying doses to mice, the CRBC phagocytosis rate and phagocytic index were significantly increased in the 0.17 g/kg/day (medium-dose) group (*p* < 0.01) and further increased in the 0.50 g/kg/day (high-dose) group (*p* < 0.001), whereas the carbon clearance capacity was significantly increased only in the high-dose group (by 13%). Compared with the control group, the high-dose group showed a significant increase of 13% in carbon clearance capacity, 33% in CRBC phagocytosis rate, and 66% in phagocytic index, respectively. These results indicate that LPB27 significantly enhanced the phagocytic function of mononuclear phagocytes in mice, with the most pronounced improvement observed in the phagocytic index, suggesting its strong non-specific immune-enhancing effect.

##### Natural Killer Cell Activity

**Figure 5:**
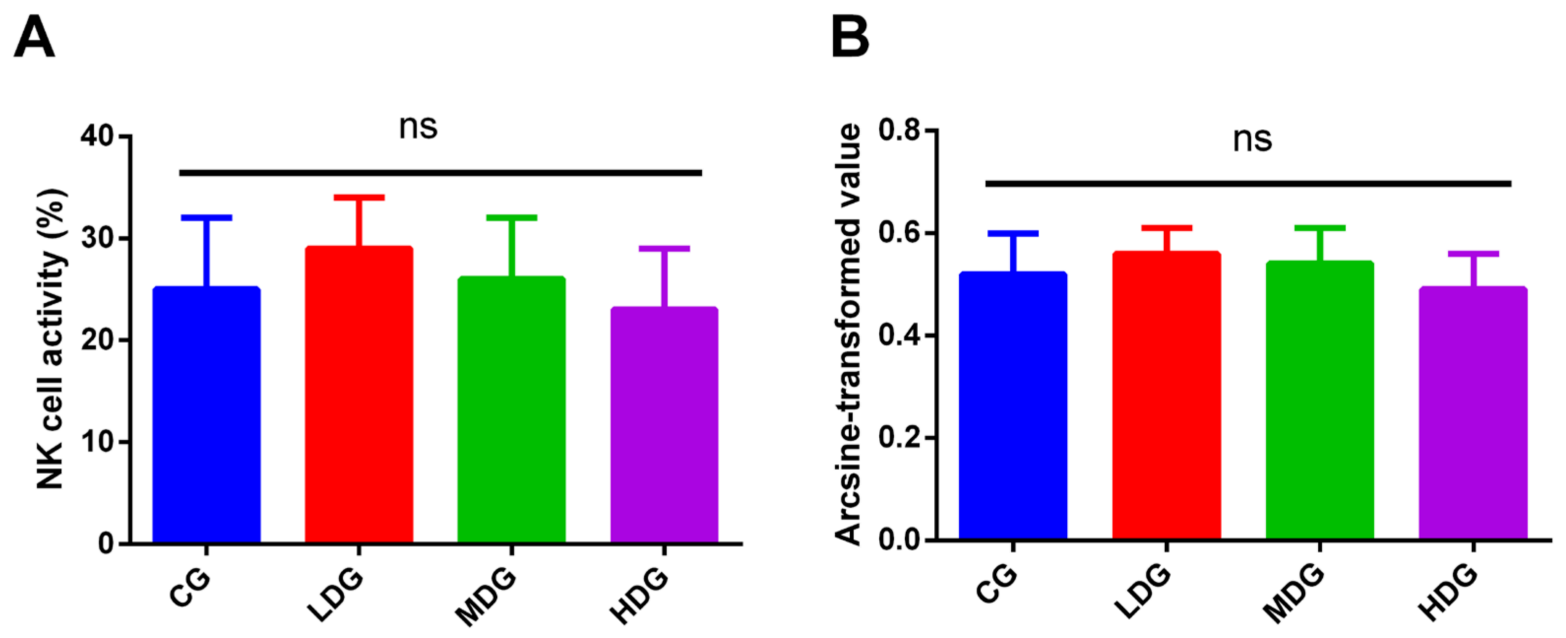
Effects of LPB27 dose on NK cell activity in mice. Note: NK cell activity (**A**) and arcsine-transformed value (**B**). CG (0 g/kg/day), LDG (0.08 g/kg/day), MDG (0.17 g/kg/day), HDG (0.50 g/kg/day). ns, not significant (*p* > 0.05). Data are expressed as mean ± SD (*n* = 12).

NK cells are a key component of the innate immune system and can directly eliminate infected, stressed, or transformed cells [28]. As shown in Figure 5, following 31 days of oral administration of different doses of LPB27 to mice, no statistically significant differences were detected among the various treatment groups. These results demonstrate that, within the evaluated dose range, LPB27 does not affect nonspecific immune function through modulation of NK cell activity.

LPB27 significantly enhanced carbon clearance capacity, macrophage phagocytosis rate, and phagocytic index, while having no apparent effect on NK cell activity, indicating that its innate immunomodulatory effect is effector-cell-selective rather than a broad activation of all innate immune components. Macrophages and NK cells differ in their activation requirements and receptor repertoires, and probiotics may preferentially stimulate the mononuclear phagocyte system through the microbiota–gut–immune axis without directly modulating NK-cell-mediated cytotoxicity.

### 3.3. 16S Analysis of Gut Microbes

To assess whether LPB27 modulates the host immune system through the gut microbiota, 16S rDNA gene sequencing was performed to analyze microbial community diversity, composition, and predicted function.

#### 3.3.1. Analysis of Microbial Community Diversity

**Figure 6:**
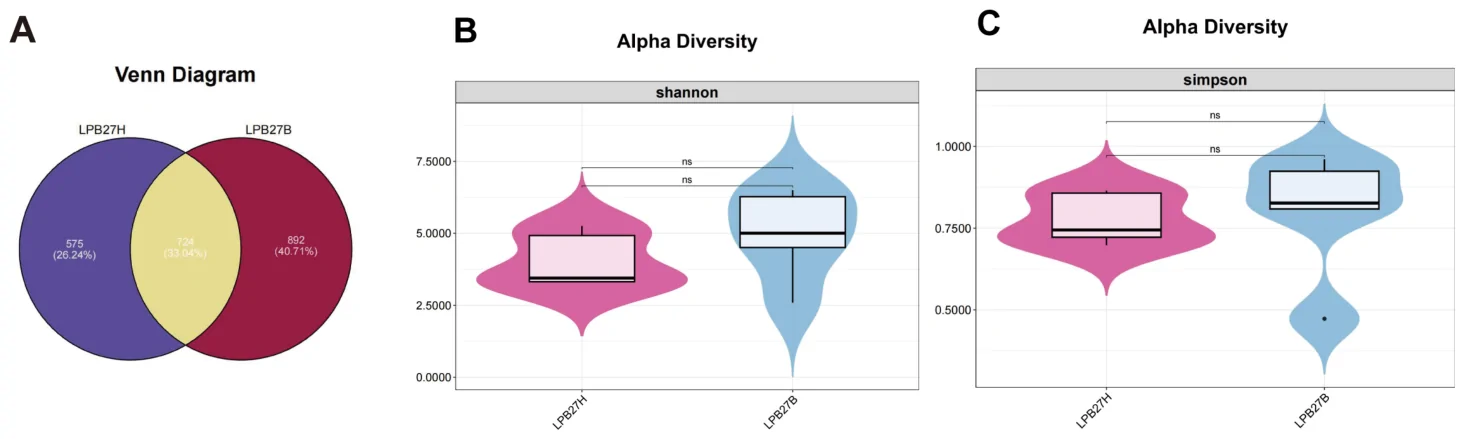
16S rDNA gene sequencing analysis of mouse fecal microbiota at day 31. Note: Venn diagram of ASVs (**A**), Shannon index (**B**), and Simpson index (**C**). LPB27B (control), LPB27H (high-dose, 0.50 g/kg/day). ns, not significant (*p* > 0.05). Data are expressed as mean ± SD (*n* = 6).

ASV analysis (Figure 6A) showed that the proportion of unique ASVs in the control group (LPB27B) and the high-dose group (LPB27H) accounted for 26.24% and 40.71% of the total sequences, respectively. These findings indicate that LPB27 intervention altered the species composition of the intestinal microbiota in mice.

Alpha diversity analysis revealed that the Shannon index (Figure 6B) and Simpson index (Figure 6C) of the high-dose group showed a declining trend compared with the control group, but no statistically significant intergroup difference was observed (*p* > 0.05).

**Figure 7:**
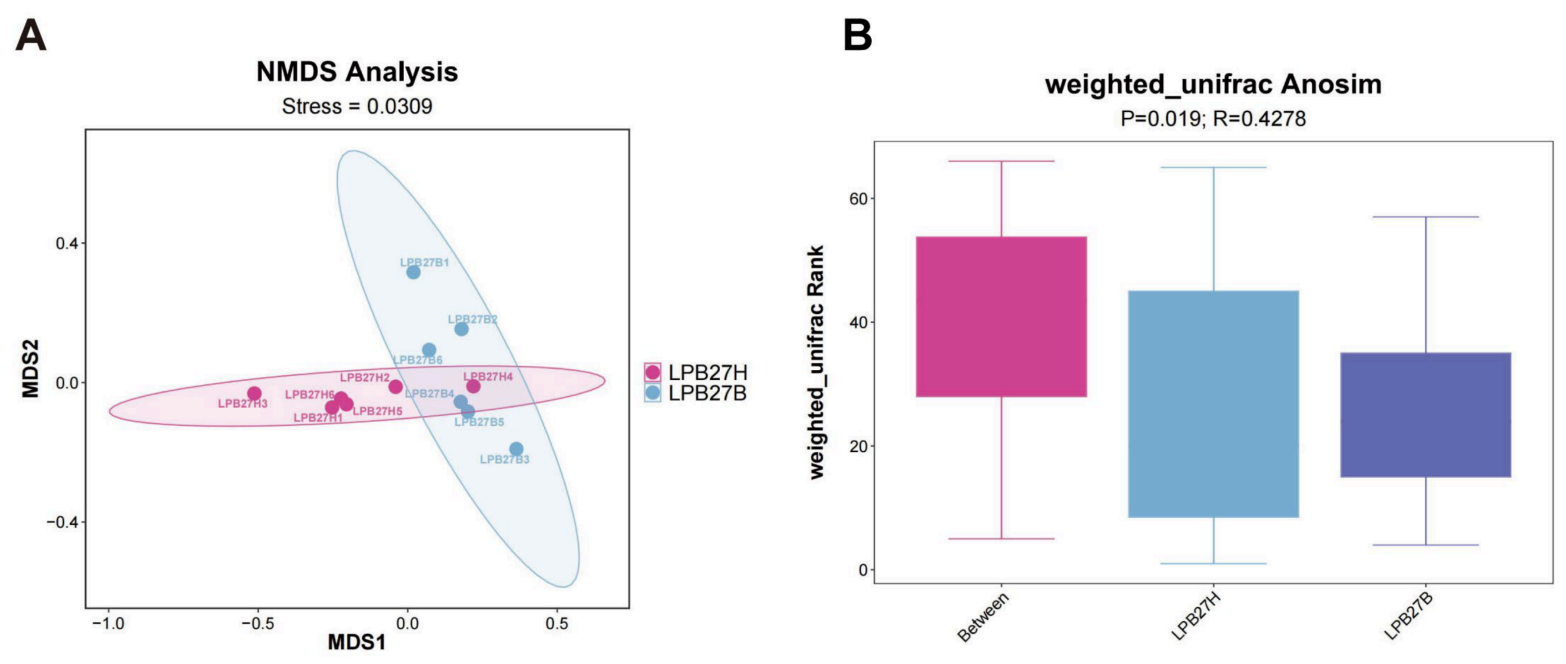
Beta diversity analysis of the mouse fecal microbiota at day 31. Note: Non-metric multidimensional scaling (NMDS) analysis (**A**) and analysis of similarities (ANOSIM) based on weighted UniFrac distances (**B**). LPB27B (control), LPB27H (high-dose, 0.50 g/kg/day).

Beta diversity analysis (Figure 7A) demonstrated clear clustering and separation between samples from the high-dose group and the control group, as shown by non-metric multidimensional scaling (NMDS). This separation was further supported by analysis of similarities (ANOSIM) (Figure 7B), which revealed that the intergroup differences were statistically significant compared with intragroup variations (*p* = 0.019, R = 0.4278).

In conclusion, although LPB27 intervention did not affect the species richness of the gut microbiota, it significantly changed the community composition structure of the gut microbiota.

#### 3.3.2. Community Composition and Differential Analysis

**Figure 8:**
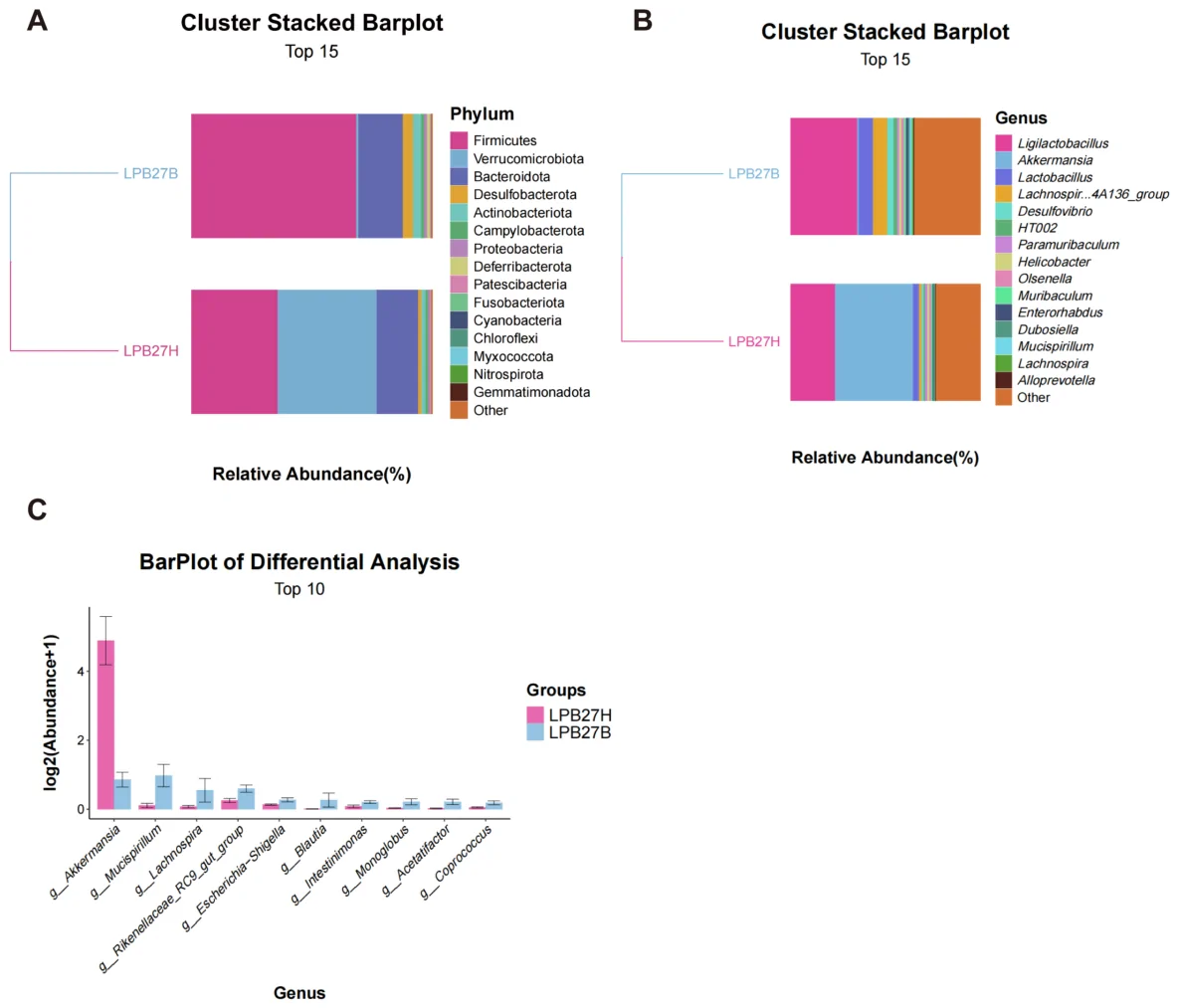
Relative abundance and differential analysis of the mouse fecal microbiota at day 31. Note: Phylum-level cluster stacked barplot (**A**), genus-level cluster stacked barplot (**B**), and differential analysis of genus abundance (**C**). LPB27B (control), LPB27H (high-dose, 0.50 g/kg/day).

To investigate the impact of LPB27 on the structural composition of the mouse gut microbiota and to assess microbial differences at the phylum and genus levels, annotation analysis was performed to identify microbial species and their relative abundances across different taxonomic levels (Figure 8A–C).

The annotation results revealed that, at the phylum level, the Firmicutes/Bacteroidota ratio was decreased and the abundance of Desulfobacterota was reduced in LPB27H group compared to LPB27B group. In contrast, the abundance of Verrucomicrobiota was significantly increased in LPB27H group. At the genus level, the abundance of *Ligilactobacillus* was decreased, whereas the abundance of *Akkermansia* was significantly elevated in LPB27H group compared to LPB27B group.

Moreover, a rank-sum test was conducted to assess statistically significant differences at the genus level. Compared with the LPB27B group, the LPB27H group showed a significantly higher abundance of the beneficial bacterium *Akkermansia*, whereas the abundance of the potentially pathogenic bacterium *Mucispirillum* was significantly reduced. These findings suggest that LPB27 primarily affects the mouse gut microbiota by reshaping its community structure, enriching beneficial bacterial genera while reducing potentially pathogenic taxa, thereby contributing to an improved gut microbial ecology that may support immune system modulation.

LPB27 administration was associated with remodeling of the gut microbial community, with beneficial genera increasing and potentially pathogenic genera decreasing, indicating an improved intestinal ecology. The enrichment of *Akkermansia* was particularly notable; this genus has been increasingly recognized as an important candidate probiotic closely linked to the modulation of host immunity [29], and its increased abundance may further support immune regulation.

#### 3.3.3. Functional Annotation Analysis Using PICRUSt2

**Figure 9:**
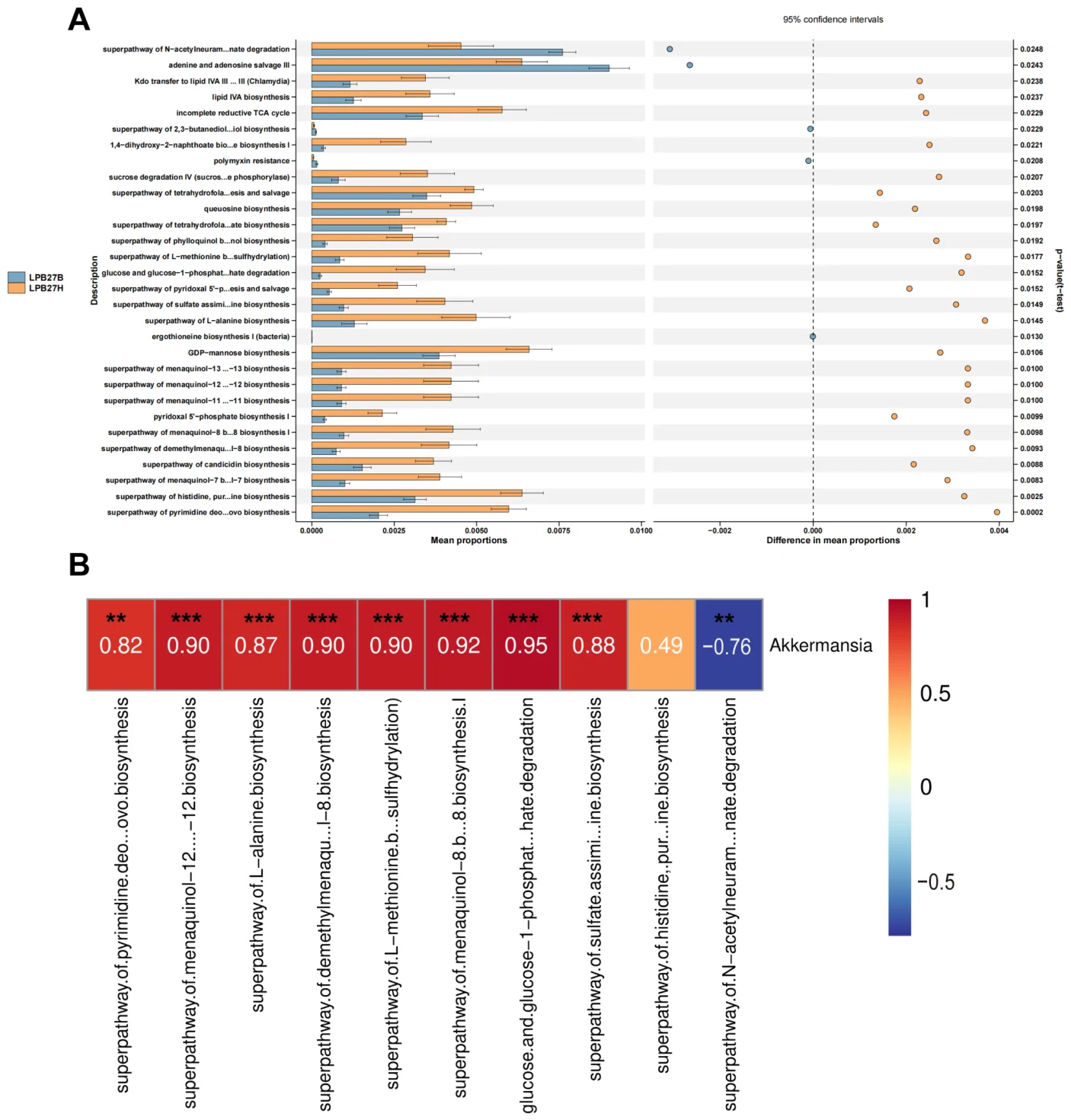
PICRUSt2-predicted functional profiling of the gut microbiota. Note: Differential abundance analysis of KEGG metabolic pathways between LPB27B (control) and LPB27H (high-dose, 0.50 g/kg/day) (**A**) and Pearson correlation between *Akkermansia* abundance and the predicted metabolic pathways (**B**). In (**A**), the left panel shows the mean relative abundance of each pathway in the two groups (blue, LPB27B; orange, LPB27H), while the right panel plots the difference in mean proportions, with the confidence interval crossing zero indicating no significant difference. ** *p* < 0.01, *** *p* < 0.001.

PICRUSt2 is a bioinformatics tool that utilizes 16S rRNA gene sequences to predict metagenomic functional profiles [30]. Based on the sequencing data, a predictive analysis of metabolic functional pathways was performed using the KEGG Pathways database. The results indicated that the administration of LPB27 was associated with the predicted upregulation of multiple metabolic processes in the high-dose group (Figure 9A), including the superpathway of pyrimidine deoxyribonucleotides de novo biosynthesis. Further analysis suggested that these predicted metabolic pathways are potentially linked to the provision of raw materials and energy necessary for the synthesis of immunoproteins [31].

Combining these findings with the previous analysis results, the abundance of *Akkermansia* was significantly increased following LPB27 administration. Based on this observation, we further analyzed the correlation between *Akkermansia* abundance and the predicted KEGG metabolic pathways using the Pearson correlation coefficient (Figure 9B). The analysis revealed that the abundance of *Akkermansia* was significantly positively correlated with several predicted metabolic pathways, including the superpathway of pyrimidine deoxyribonucleotides de novo biosynthesis, whereas it was negatively correlated with the superpathway of N-acetylneuraminate degradation. These findings suggest that *Akkermansia* may regulate immune function through these predicted metabolic pathways.

Notably, PICRUSt2 infers functional profiles from 16S rRNA gene sequencing data rather than directly measuring metagenomic activity [32], so the predicted pathway abundances reflect only potential functional capacity and should be interpreted as correlational rather than causal evidence. Although the abundance of *Akkermansia* correlated positively with several predicted pathways, including the pyrimidine deoxyribonucleotide biosynthesis pathway, suggesting that this genus may participate in regulating the synthesis of immune-related proteins, the inherent limitations of this predictive approach preclude establishing a causal relationship, or its direction, from the present data.

### 3.4. Analysis of the Intestinal Environment in Mice After 15 Days of Administration

At day 31, although gut microbial diversity showed no significant change, the community structure was significantly altered, with the abundance of *Akkermansia* exhibiting a significant increase. To explore the dynamics of the gut microbiota during the administration period, we further analyzed the short-chain fatty acid levels and the changes in bacterial genera in fecal samples collected at day 15 of intervention.

#### 3.4.1. Determination of SCFAs

**Figure 10:**
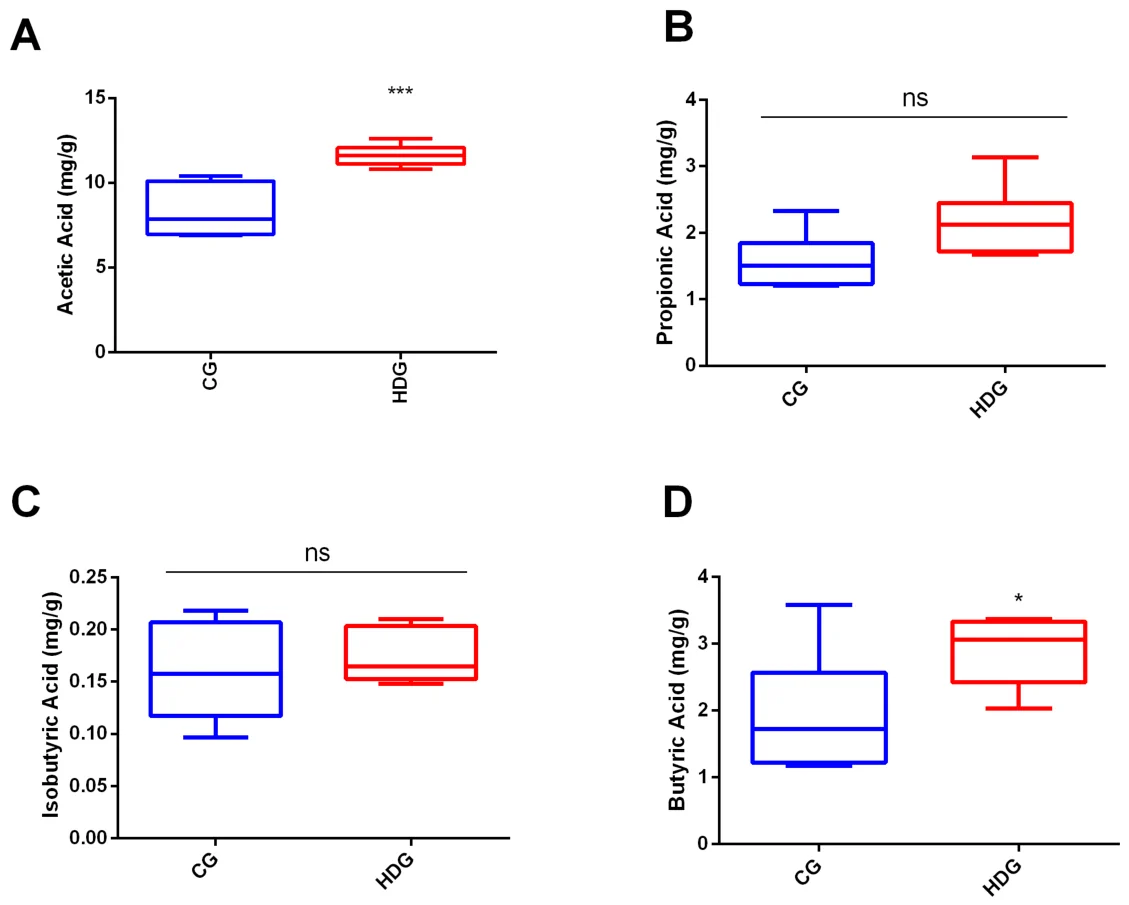
Concentrations of short-chain fatty acids in mouse feces. Note: Acetic acid content (**A**), propionic acid content (**B**), isobutyric acid content (**C**), and butyric acid content (**D**). CG (0 g/kg/day), HDG (0.50 g/kg/day). ns *p* > 0.05, * *p* < 0.05, *** *p* < 0.001 vs. CG. Data are expressed as mean ± SD (*n* = 12).

Short-chain fatty acids (SCFAs), together with the branched-chain fatty acid (BCFA) isobutyric acid, are key indicators of the intestinal microenvironment. As illustrated in Figure 10, following 15 days of LPB27 administration, the concentrations of acetic acid and butyric acid in the mouse intestine were significantly increased (Figure 10A–D). This observation suggests a marked enhancement of intestinal homeostasis, which may facilitate the proliferation and colonization of beneficial gut microbiota. Indeed, SCFA-producing taxa such as *Lactobacillus*, *Muribaculum*, and *Lachnospiraceae_NK4A136_group* were enriched at day 15, which likely contributed to the early elevation of acetic and butyric acid at this time point.

#### 3.4.2. Community Composition and Differential Analysis at Day 15

**Figure 11:**
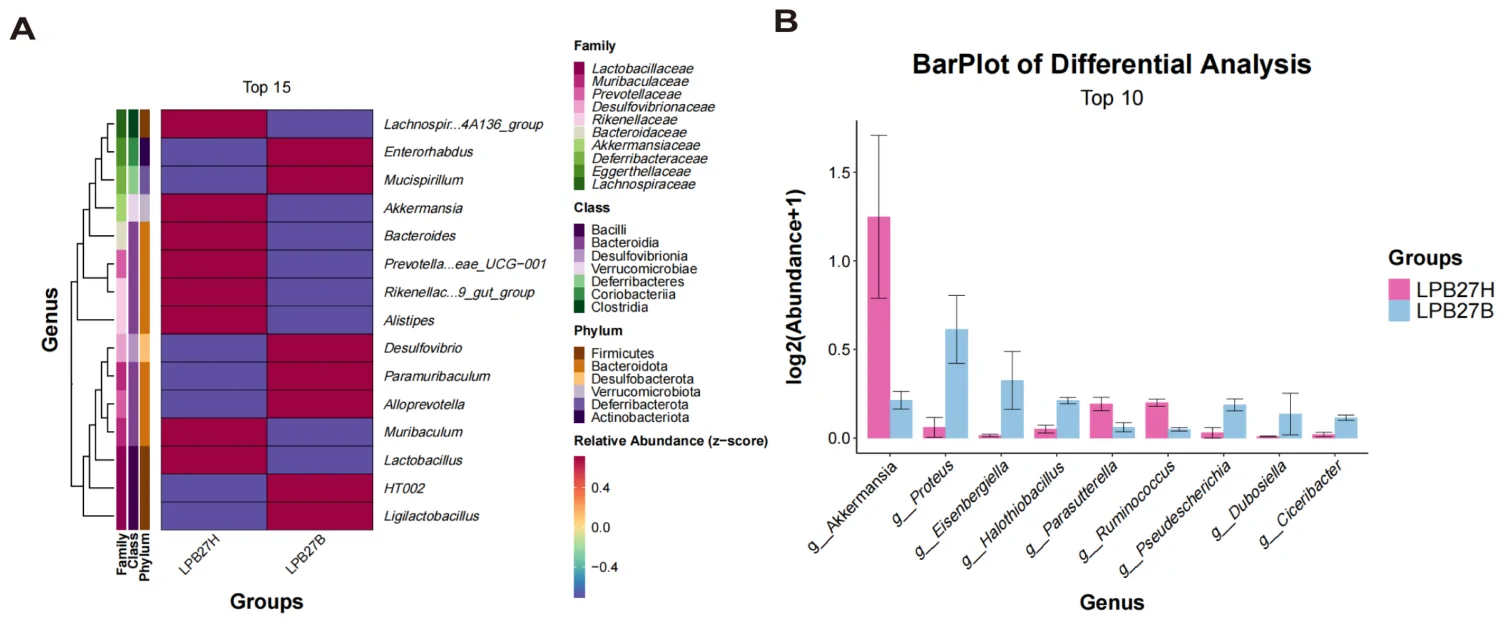
16S rDNA gene sequencing analysis of mouse fecal microbiota at day 15. Note: Genus-level heatmap of relative abundance (**A**) and barplot of differential genus analysis (**B**). LPB27B (control), LPB27H (high-dose, 0.50 g/kg/day).

16S rDNA sequencing analysis revealed that after 15 days of LPB27 administration, at the genus level, the abundances of several beneficial bacterial taxa, including *Lactobacillus*, *Muribaculum*, *Lachnospiraceae_NK4A136_group*, and *Akkermansia*, were increased, whereas the abundances of potentially pathogenic genera such as *Desulfovibrio* and *Mucispirillum* were decreased. Differential analysis confirmed the enrichment of these beneficial taxa, among which SCFA-producing genera such as *Lactobacillus* and *Lachnospiraceae_NK4A136_group* were prominent. Compared with the gut microbiota composition observed on day 31, the trend toward enrichment of beneficial taxa was already apparent as early as day 15. These results suggest that the beneficial remodeling of the gut microbiota began within 15 days of administration and was further consolidated by day 31, with a sustained shift away from potentially pathogenic taxa (Figure 11A,B). Both fecal sampling time points (days 15 and 31) fell within the 31-day administration period; no samples were collected after the treatment period ended, so microbial changes beyond day 31 were not assessed in this study.

Gut microbiota analyses at days 15 and 31 after administration indicate that LPB27 intervention reshaped the intestinal microbial ecosystem early in the treatment period, creating a microenvironment favorable for the proliferation of *Akkermansia*. With prolonged intervention, *Akkermansia* showed a sustained and significant enrichment trend.

### 3.5. Measurement of IgA, IgG, and Serum Inflammatory Markers

**Figure 12:**
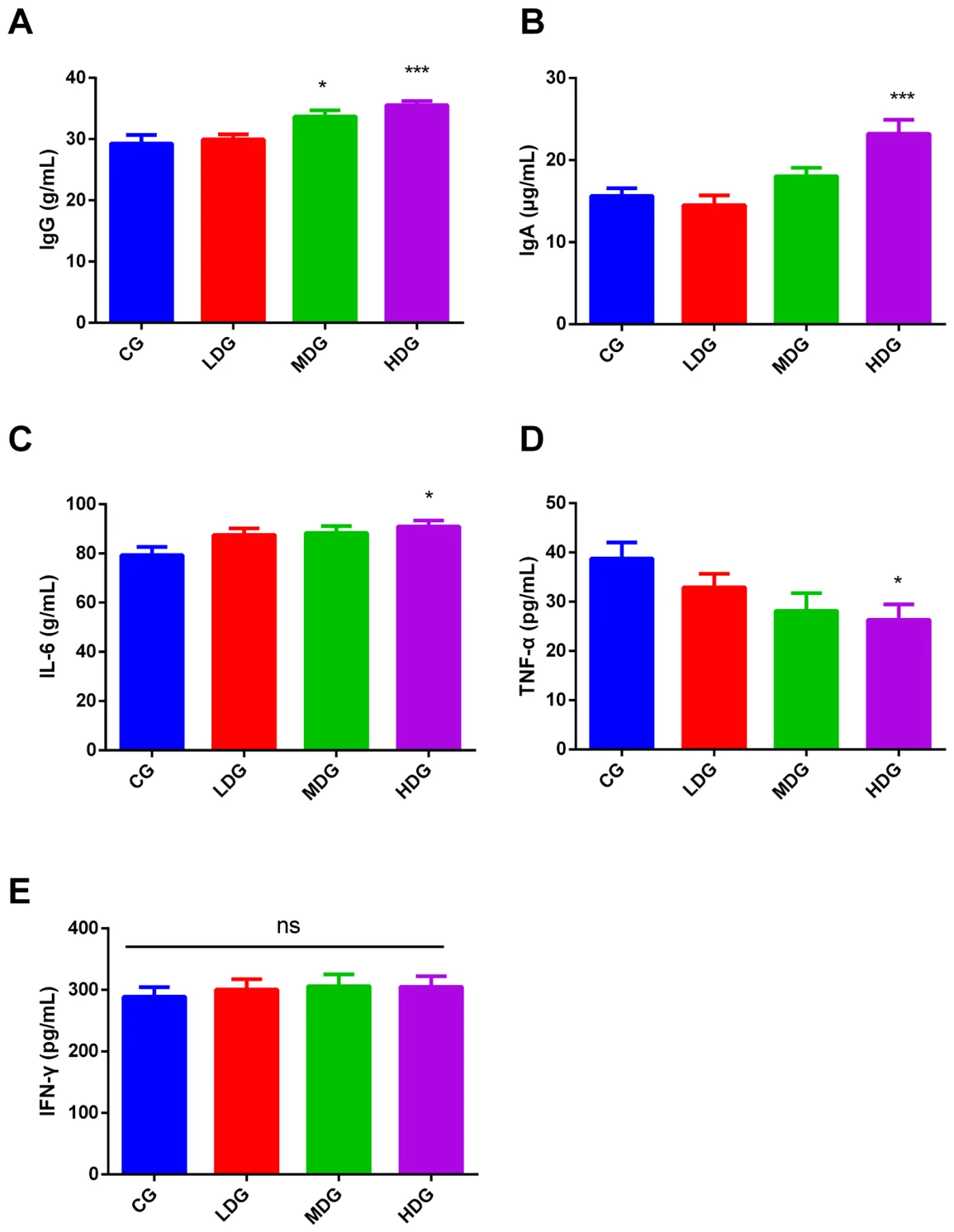
IgA, IgG and serum inflammatory indicators. Note: Serum IgG concentration (**A**), Serum IgA concentration (**B**), Serum IL-6 concentration (**C**), Serum TNF-α concentration (**D**), Serum IFN-γ concentration (**E**). CG (0 g/kg/day), LDG (0.08 g/kg/day), MDG (0.17 g/kg/day), HDG (0.50 g/kg/day). ns *p* > 0.05, * *p* < 0.05, *** *p* < 0.001 vs. CG. Data are expressed as mean ± SD (*n* = 12).

The 16S gene sequencing-based prediction suggested that the immune-modulating effect of LPB27 might be closely related to its ability to enrich beneficial gut bacteria, thereby activating the pathways for the supply of raw materials and energy required for immune-related protein synthesis. Therefore, further measurements of immunoglobulin and serum inflammatory factor concentrations in mice were conducted for verification (Figure 12).

As shown in Figure 10, after 31 days of oral administration of different doses of LPB27 to mice, the serum levels of IgA, IgG, and IL-6 in the 0.50 g/kg/day (high-dose) group were significantly increased compared with those in the control group, whereas the serum level of TNF-α was significantly decreased. These data suggest that LPB27 supplementation is associated with increased abundance of immune-related proteins in the body.

### 3.6. Limitations

This study has several limitations. The molecular mechanisms underlying LPB27-mediated gut microbiota remodeling remain unclear, and the functional predictions were inferred from the PICRUSt2 algorithm; the limited species/strain-level resolution of 16S rRNA gene sequencing constrains the interpretation of the predicted pathways. In addition, the causal interactions among LPB27, the gut microbiota, and the host remain to be systematically resolved.

## 4. Conclusions

In healthy mice, oral administration of LPB27 at 0.50 g/kg/day enhanced adaptive immunity, including delayed-type hypersensitivity, lymphocyte proliferation, hemolytic plaque formation, and HC50, together with innate immunity assessed by carbon clearance and macrophage phagocytosis. Concurrently, serum IgA, IgG, and IL-6 levels increased while TNF-α decreased. In the intestinal environment, LPB27 reshaped the gut microbial community, significantly enriching immunomodulation-associated genera, particularly *Akkermansia*, while reducing potentially pathogenic taxa and raising fecal acetate and butyrate concentrations. These findings suggest that LPB27 may enhance host immunity by optimizing the intestinal microenvironment and enriching immune-related genera. Overall, LPB27 represents a promising candidate probiotic warranting further investigation of its immunomodulatory potential.

## Data Availability

Parts of the dataset contain preliminary data for our follow-up unpublished research. Therefore, complete raw data can only be obtained by contacting the corresponding author.
